# Moving from psychiatric practice in the UK to Australia: some personal reflections

**DOI:** 10.1192/bjb.2024.69

**Published:** 2025-06

**Authors:** Graham Walker, Andrew Carroll

**Affiliations:** 1 University of Glasgow, UK; 2 NHS Scotland, Glasgow, UK; 3 Swinburne University of Technology, Victoria, Australia

**Keywords:** Australia, registrar, reflection, comparison, career

## Abstract

In this article, we reflect on factors which may tempt psychiatrists to move from working in the UK to Australia. A comparison between the UK and Australian healthcare systems is presented. Following this, G.W. offers personal reflections on his transition between working in the UK and Australia, including an experience of being a patient, the benefits of working and training in the respective countries, and personal sacrifices which must be considered. We conclude that individual clinicians must weigh up the positives and negatives of the system which they want to work within, with the best option for each person being specifically individual to them.

## Perceived career opportunities

A recent literature review identified themes which contributed to a positive experience and potentially career-influencing interest in psychiatry during prevocational training years. These included provision of mentorship and consistent, high-quality supervision, under the support of a consultant who was supportive, responsive and interested in the profession. Striking the balance between supported autonomy with a reasonable degree of supported responsibility as per trainee skill levels was also seen to be important. Witnessing patient recovery across a variety of treatments, clinical presentations and locations was also associated with job satisfaction.^1^

Recent research^2^ suggested that in comparison with their colleagues in the UK, doctors in Australia are slightly more engaged and view their work more positively. Good interpersonal relationships were found to be the only variable on which UK doctors scored more positively than their Australian counterparts. Findings suggested that overall, doctors in Australia felt valued and empowered, had purpose and direction and worked in a collaborative culture. It was suggested that there may be numerous factors that influence how engaged doctors are in both countries: the most prominent of these appeared to be working conditions and lifestyle, driven by funding and other economic issues.^2^

Psychiatrists are increasingly attracted by work outside the UK. Reasons given for wishing to leave practice in the UK include gaining wider experience, a belief that things would be ‘better’ elsewhere and a negative view of the National Health Service (NHS) and its culture, state and politics.^3^ Other reasons cited have included better training or job opportunities, better pay and conditions, family reasons and higher career expectations.^3^ An increasingly negative view is held by many doctors of many aspects of the experience of being a junior doctor in the NHS and the difficulty of delivering high-quality patient care within what many see as an under-funded system.^3^ Conversely, consultant posts in Australia promise excellent salaries, flexible working patterns and extensive well-being initiatives.^4^

## Comparison of healthcare in the UK versus Australia

In the UK, the NHS embodies the essence of universal healthcare, offering free consultations and treatment at the point of use. General practitioners (GPs) serve as the lynchpin, orchestrating referrals and coordinating patient journeys through the complex web of healthcare services.^5^ In Australia, the landscape is characterised by more of a mixed model, with primary care facilitated through both public and private providers. GPs operate independently or within private clinics, fostering a system that provides citizens with choices in their healthcare journey. Accessibility is maintained, but the balance between public and private provision introduces an element of market dynamics that is less evident in the UK. In Australia, those who earn above a certain threshold are incentivised by the tax system to purchase private healthcare insurance, via the private healthcare insurance rebate.^6^ This takes pressure off of the public system; as a result, healthcare waiting lists in Australia are generally shorter.^7^ The Australian federal government sets a specific reimbursement for each medical intervention from psychology appointments to specific surgeries, via Medicare. This amount is reasonable but can be privately topped up either by individual payments and/or via private health insurance.^8^

By contrast, the NHS in the UK operates on the principles of equality, with the belief that health should not be a commodity. The idea of ‘free at the point of use’ is deeply ingrained, ensuring that a person's financial status does not impede their access to healthcare. Although this eliminates disparities in care based on income, it also places a considerable burden on the NHS to manage resources efficiently. There is significant public opinion in support of the concept of the NHS, and any political suggestion of widespread reform is generally not a vote winner.^9^ Therefore, despite the fact that the NHS appears to be struggling to keep up with current demands, widespread changes do not appear to be forthcoming any time soon.

## Background on the author's journey

Before commencing specialty child and adolescent psychiatry training in the UK in August 2023, G.W. took a 3-year break from UK practice to work as a senior registrar in adult forensic psychiatry in Melbourne, Australia. Although he was not a formal College-accredited trainee, he was treated similarly to an Australian psychiatry trainee and worked in 6-month placements across a wide variety of forensic psychiatry settings. This gave him a unique experience of psychiatric practice in another area of the world. A.C. is a consultant psychiatrist who provided clinical supervision to G.W. across an 18-month period and shared many hours of reflection discussing matters raised in this paper.

The following section of this paper has been written in the first person, emphasising that this is G.W.'s personal experience. We acknowledge that others may differ in their opinion of these reflections. In addition, as the author only worked within one service and one state (Victoria), the experience of working in alternative services may differ. Both authors are also citizens of the UK.

## Author's personal reflections

Before considering my professional experience of working as a psychiatrist in Australia, I should consider my own experience of being a patient. When living in Australia, I had to have a minor investigative medical procedure performed with specialist involvement. I was able to get an appointment with my general practitioner (GP) within a day, and after appropriate investigation was referred to and communicated with a specialist within a week. I then had the investigative procedure completed within a 2-week period. When I returned to the UK, I was recommended to link in with the UK's equivalent specialist for appropriate follow up. As would be expected, I requested referral for this via my GP. I had to wait 3 weeks for the first available GP appointment and have yet to receive an appointment for specialist review despite having waited 10 months at this point. I know of multiple other friends and family members who have been waiting a similar amount of time for specialist investigation, with some having to resort to expensive private care without any government funding so they can be seen in a timely fashion. In Australia, I could pay a comparatively small fee to have this procedure performed in a private setting, with Medicare covering all other fees.

A further reflection on Australian provision of services is that of location. Owing to the expansive geography of Australia, the population is generally situated in a small number of large cities, mostly on the east coast. This is unlike the UK, where the population is much more equally distributed across the country. As a result, healthcare services in Australia tend to be much more centrally located, and a single service may cover a full state which is geographically larger than the UK as a whole. As a result, patients may have to travel much larger distances for clinics, or, if requiring admission, be much further from their home. However, I did observe effective use of telehealth provision, which could help reduce such limitations.

There do appear to be some negatives to having higher numbers of services providers in Australia, with less readily connected information sharing. This includes an increased risk in the phenomenon of ‘prescriber shopping’, where owing to fewer shared electronic records of prescriptions and previous diagnoses, some patients may be able to get prescribed treatments that may not have been issued if the pharmacist was aware of their full history – for example, the risk of diversion of controlled medications. However, the risk of this is being reduced by the roll out of SafeScript, a clinical tool that provides access to a patient's prescription history for high-risk medicines to enable safer clinical decisions.^10^ As a clinician, I also found it harder to gather information on a patient's previous treatment, for example, their previous blood results or prescriptions if these were documented at another service.

Moving on to more psychiatry-specific differences, I had to adapt to differing approaches to coercive care. One example is the more common use of mechanical restraint in emergency department settings; this is perhaps required more pressingly owing to the increased prevalence of illicit methamphetamine use, which can cause vulnerable individuals to present in a highly agitated manner. There is also a difference in medication preferences, for example, the widespread use of the antipsychotic medication droperidol – which I had never seen prescribed in the UK – in emergency departments across Australia. It must be noted that practice varies across health organisations, and mental health law also varies between the states of Australia.

As noted above, economic rewards are among the main draws for healthcare professionals to work in Australia. Personally, since moving back to work in the UK, I have taken a basic salary cut of approximately 38%. It must be noted, however, that in major cities such as Sydney and Melbourne, property is significantly more expensive than in most of the UK. I believe that one reason that Australian public sector healthcare workers’ salaries are higher is that there is more option for private work, and therefore public healthcare providers must offer more incentive to draw in employees, unlike the relative monopoly of the NHS. It is thus interesting that some UK-based healthcare staff will adamantly argue for an increased salary while also being supportive of the current structure of the NHS, which is perhaps one reason salaries cannot increase without major change.

Another difference I noted was a difference in how on-call shifts are remunerated. In the UK, clinicians are paid a banding supplement based on an average of how many on-calls are worked by each doctor on the rota. Based on random chance, some doctors on the rota can work more on-call shifts than their colleagues for the same salary. In Australia, clinicians are paid per on-call shift. The rate per on-call is significantly higher than a UK locum rate. As a result, it appeared that covering on-call shifts was more desirable.

While in Australia, I worked with highly skilled colleagues from across the world. Despite this, I noted that psychiatrists who were UK trained were highly valued by Australian services. I also noted that, on average, UK-trained doctors had very high skill and knowledge levels. In relation to training opportunities, my opinion is that there may be some benefits of training in psychiatry in the UK over Australia. The UK psychiatry training system appears to be more structured, with more opportunities to develop leadership skills. Part of the reason for this is that there is less of a distinction between more and less experienced trainees, and the equivalent of core trainees and registrars are all classed as ‘registrars’. Therefore, there is less of a system where psychiatry registrars provide on-call supervision to core trainees, as in the UK. Despite this, the training overall appears to be relatively similar, and from a day-to-day work perspective, it felt intuitive to transfer between working in each healthcare system.

In terms of availability of reflective practice for psychiatry trainees working in Australia, it appears that this is less structured and up to the individual health services to organise, rather than being enshrined in the curriculum. It is part of the core learning curriculum of the (UK) Royal College of Psychiatrists for psychiatry trainees that trainees will attend regular reflective practice groups during their training.^11^ Usually, this requirement is achieved by weekly attendance at reflective practice groups that typically model Balint principles for doctor–patient discussions. Before a UK psychiatry trainee is permitted to start providing supervised psychological therapy (also a requirement of training), the trainee must have attended a regular reflective practice group for a period of at least a year.^11^ There is no such stated curriculum requirement within the RANZCP (Royal Australian and New Zealand College of Psychiatrists) learning outcomes.^12^

Despite the above, I believe that there are other areas in which the Australian training scheme may serve its trainees better. One example is the length of time spent in generalist training after medical school graduation. In the UK, this foundation training lasts 2 years, whereas in Australia the ‘internship’ lasts 1 year. In such training years in both countries, trainees have more of a chance of being placed in a specialty for a 4–6-month placement in which they have no major interest. It therefore may be of benefit for the trainee if they can more promptly start working in the specialty they want to work in. However, a counter argument would be that working in an alternative specialty for an additional period can make one a more rounded doctor with skills that can be taken into another specialty.

Another difference in entering the respective psychiatry training programmes is that in the UK, applicants must apply nationally and then be allocated to a specific health board and region, which are ranked in order of preference. There is a chance that trainees could be allocated somewhere a significant distance from their current abode, requiring relocation; this can apply even if they are placed in the region where they already live, as more peripheral hospitals can be several hours drive from central locations in the health region. In Australia, candidates instead apply directly to hospitals in which they would be willing to work. This offers more flexibility, as the trainee can apply to work in a different hospital in another area if their situation changes.

Further along in one's training, another benefit of the Australian training system is the ability to complete an Advanced Certificate in a psychiatric subspecialty while working on the job as a consultant. This would be the equivalent of a Certificate of Completion of Training in the UK, which can only be obtained at the lower-paid registrar grade. Another difference is the continued medical education budget – in Victoria, the consultant pay deal stipulates that consultants working in the public sector can claim a generous study allowance to use on equipment (including books or laptops), research or travelling to conferences. The amounts vary between states but are substantially higher than funding for such activities available to UK consultants.

It can be a challenge to transfer experience for accreditations between countries. My understanding was that as I had not reached consultant level in the UK, if I was to transition into the RANZCP training programme, I would probably need to sit and pass all of their exams, despite these appearing to be similar to the Royal College of Psychiatrists qualifications which I have already obtained. I would also need to satisfy several other competencies, for example, completion of a supervised psychotherapy case and submission of a scholarly research project, despite already having a good level of experience in such areas. Although these are clearly important competencies to have as a psychiatrist, I did not feel it was best for my personal development to have to ‘jump through hoops’ like this, and I therefore elected to move back to the UK to finalise my psychiatric training. It does appear that the process of applying to RANZCP via comparability assessment when one has reached consultant level may be an easier process to navigate.^13^

My final reflection is that of the non-work-based negatives of working in Australia, if not born in Australia. Australia is an amazing country, with amazing landscapes and people, and so much to see and do. Despite this, it is almost as far as can be from the UK, with a resulting absence from family and childhood friends. This is the main factor that appears to bring people back to the UK after having worked in Australia, and personally I know that since coming back to the UK I am really appreciating this proximity again. This is one example of an important dynamic which needs to be considered when considering one's own professional well-being.

## Conclusion

As we reflected on the differences between the UK and Australian healthcare systems, we were reminded that there is no one-size-fits-all approach to healthcare. Both systems grapple with challenges unique to their structures, and the quest for an ideal healthcare model remains an ongoing journey, shaped by the ever-evolving needs and expectations of the societies the systems serve. It is important that clinicians continue to develop their professional skills within their training years and beyond as consultants, taking in information from all available avenues and considering best practice to model from the various settings in which they have worked. Individual clinicians must balance the positives and negatives of wherever they chose to base themselves, to make the best decision to satisfy their own individual professional and, even more so, personal needs.

## About the authors

**Graham Walker** is a child and adolescent psychiatry registrar, working in NHS Scotland, and a clinical lecturer in child and adolescent psychiatry, based in the School of Health & Wellbeing at the University of Glasgow, UK. **Andrew Carroll** is a consultant forensic psychiatrist and adjunct associate professor in the Centre for Forensic Behavioural Science, Swinburne University of Technology, Australia.
